# A prospective study of lifetime physical activity and prostate cancer incidence and mortality

**DOI:** 10.1038/sj.bjc.6605404

**Published:** 2009-10-27

**Authors:** N Orsini, R Bellocco, M Bottai, M Pagano, S-O Andersson, J-E Johansson, E Giovannucci, A Wolk

**Affiliations:** 1 Division of Nutritional Epidemiology, The National Institute of Environmental Medicine, Karolinska Institutet, Stockholm, Sweden; 2 Department of Statistics, University of Milano-Bicocca, Milan, Italy; 3 Department of Epidemiology and Biostatistics, Arnold School of Public Health, University of South Carolina, Columbia, SC, USA; 4 Department of Biostatistics, Harvard School of Public Health, Harvard University, Boston, MA, USA; 5 Department of Urology, University Hospital Örebro, Örebro, Sweden; 6 Department of Epidemiology, Harvard School of Public Health, Harvard University, Boston, MA, USA

**Keywords:** lifetime, occupational activity, walking, cycling, physical activity, cohort, prostate cancer

## Abstract

**Background:**

The possible benefit of lifetime physical activity (PA) in reducing prostate cancer incidence and mortality is unclear.

**Methods:**

A prospective cohort of 45 887 men aged 45–79 years was followed up from January 1998 to December 2007 for prostate cancer incidence (*n*=2735) and to December 2006 for its subtypes and for fatal (*n*=190) prostate cancer.

**Results:**

We observed an inverse association between lifetime (average of age 30 and 50 years, and baseline age) total PA levels and prostate cancer risk. Multivariate-adjusted incidence in the top quartile of lifetime total PA decreased by 16% (95% confidence interval (CI)=2–27%) compared with that in the bottom quartile. We also observed an inverse association between average lifetime work or occupational activity and walking or bicycling duration and prostate cancer risk. Compared with men who mostly sit during their main work or occupation, men who sit half of the time experienced a 20% lower risk (95% CI=7–31%). The rate ratio linearly decreased by 7% (95% CI=1–12%) for total, 8% (95% CI=0–16%) for localised and 12% (95% CI=2–20%) for advanced prostate cancer for every 30 min per day increment of lifetime walking or bicycling in the range of 30 to 120 min per day.

**Conclusions:**

Our results suggest that not sitting for most of the time during work or occupational activity and walking or bicycling more than 30 min per day during adult life is associated with reduced incidence of prostate cancer.

An expert panel from the World Cancer Research Fund/American Institute for Cancer Research has reported that physical activity (PA) may specifically reduce the risk of advanced or aggressive prostate cancer, but no formal judgment was made regarding the strength of the evidence (WCRF/AICR, 2007). A review of 24 studies of PA and prostate cancer risk found the evidence to be inconsistent, mainly because of the heterogeneous nature of neoplasm, and urged an investigation of PA at different ages as a potentially productive approach (Friedenreich and Thune, 2001).

Two case–control studies have included a measure of lifetime PA (Friedenreich *et al*, 2004; Wiklund *et al*, 2008); one found a non-significant decrease in prostate cancer risk for lifetime recreational activity (Friedenreich *et al*, 2004), whereas the contrary was found in the other study (Wiklund *et al*, 2008). No cohort study has examined detailed measures of lifetime PA in relation to prostate cancer incidence or mortality.

To investigate lifetime total PA – and more specifically work or occupational activity and walking or bicycling, the main component of active living – in relation to incident and fatal prostate cancer, we analysed data from a population-based cohort of middle-aged and elderly men who reported their usual PA in the previous year, at age 50 and 30 years.

## Materials and methods

The population-based cohort of Swedish men was established in 1997–1998, when all eligible men (*n*=100 303) aged 45–79 years residing in Västmanland and Örebro counties (central Sweden) received an invitation to participate in the study, along with a self-administered questionnaire. This covered walking or bicycling; current waist, hip and height measurements; education level; cigarette smoking; alcohol consumption; diabetes; family history of prostate cancer; and other lifestyle factors. A total of 48 645 men returned the questionnaire.

In this analysis, we excluded participants who returned an incomplete questionnaire (*n*=92), died before 1 January 1998 (*n*=55), moved out of the study area (*n*=19) or had a previous cancer diagnosis (*n*=2592); after exclusions, 45 887 men were included. This large population-based cohort is representative of Swedish males aged 45–79 years, in terms of age distribution, educational level and prevalence of overweight (Norman *et al*, 2002). Incidence rates are also comparable; for example, the incidence rate among men aged 65–69 years is 603 in the cohort and 595 per 100 000 men in entire Sweden (NBHW, 2000).

Information on usual PA levels at different ages (current, age 50 years and age 30 years) was collected using five questions relating to occupation, housework, walking or cycling, leisure-time exercise and inactive leisure time (e.g., watching TV or reading). There were six predefined activity levels for occupational activity (from mostly sitting down to heavy manual labour) and five to six predefined categories for time spent on different activities, such as walking or bicycling (hardly ever to more than 90 min per day), home or household work (less than 1 h to more than 8 h per day), inactive leisure time (from 2 h per day or less to 5 h per day or more) and active leisure-time exercising (from less than 1 h to more than 5 h per week). There was also an open question regarding the number of sleeping hours per day.

Physical activity levels for specific activities were estimated by multiplying reported duration (hours per day) by absolute intensity. This was determined by the rate of work being performed and does not take into account the physiological capacity of the individual. The absolute intensity of activities, defined in multiples of the metabolic equivalent (MET, kcal kg^−1^ × h) of sitting quietly for 1 h, was based on a compendium of PAs (Ainsworth *et al*, 2000). More details regarding the calculations and assigned intensity values used can be found elsewhere (Norman *et al*, 2001). The total PA scores at age 30 and 50 years, and at baseline age, were calculated by summing the products of duration and intensity for each PA type. The major contributor (60%) to active leisure time was walking or bicycling.

The PA questions were validated using two 7-day activity records that were maintained 6 months apart in a group of Swedish men aged 44–78 years. The 7-day activity records were shown to correlate well with the total PA questionnaire data, with a Spearman correlation coefficient of 0.6. The reproducibility of total PA, as reflected by the Spearman correlation coefficient between the first questionnaire and that obtained 6 months later, was 0.65 (Norman *et al*, 2001).

The adult lifetime average total PA (MET, h per day), work or occupational activity level and walking or bicycling (min per day) were estimated for each participant, with at least two observed values as the average of the three measures of PA at ages 30 and 50 years, and at baseline age.

Incident cases of prostate cancer were ascertained by computerised record linkage with the Swedish National Cancer Register and the Regional Cancer Register covering the study area, both of which are estimated to be almost 100% complete (Mattsson and Wallgren, 1984). From 1 January 1998 through to 31 December 2007, during 416 172 person-years, we documented 2735 newly diagnosed cases. Information on tumour–node–metastasis stage, the Gleason grade and the value of prostate-specific antigen (PSA) at prostate cancer diagnosis were available from the Swedish Prostate Cancer Quality Registry. Incident cases were classified by subtype as localised (T1–2, NX-0, MX-0 or PSA<20 or Gleason grade⩽7) and advanced (>T2, NX-1, MX-1 or PSA>100 or Gleason grade>7). From 1 January 1998 through to 31 December 2006, during 377 904 person-years, we documented 1098 localised and 970 advanced cancers at diagnosis, 190 of which were fatal. Information on prostate cancer death was ascertained through linkage to the Swedish Register of Death Causes at the National Board of Health and Welfare. Classification of deaths was based on International Classification of Diseases (ICD-10, code 61 for prostate cancer).

### Statistical analysis

The Cox proportional hazards model was used to estimate prostate cancer rate ratios (RRs) and 95% confidence intervals associated with lifetime average PA (total daily score, work or occupational and walking or bicycling) levels.

For incidence analyses, each participant accrued follow-up time from 1 January 1998 until the date of prostate cancer diagnosis, death from any cause or study end (31 December 2007 for total prostate cancer or 31 December 2006 for subtypes of prostate cancer), whichever came first. For fatal prostate cancer analysis, each participant accrued follow-up time from 1 January 1998 until the date of prostate cancer death, death from any cause or study end (31 December 2006), whichever came first.

In all multivariate analyses, we adjusted for baseline age, waist-to-hip ratio, height, diabetes, alcohol consumption, smoking status, years of education, total energy intake, consumption of dairy product and red meat and parental history with respect to prostate cancer.

We checked whether the proportional hazard assumption was reasonable in the multivariate models. Scaled Schoenfeld's residuals were regressed against survival time. There was no evidence of departure from the assumption. Restricted cubic splines (three knot positions corresponding to quartiles of observations) were used to flexibly model and graph the multivariate-adjusted rate ratio for lifetime average total daily PA and walking or bicycling duration in predicting prostate cancer incidence and mortality. We examined potential effect modification for the relationship between lifetime total PA and total cancer incidence according to the interval between study entry and diagnosis, baseline age (⩽60, >60 years), educational level (post-secondary education *vs* lower) and waist–hip ratio (<0.95, ⩾0.95), and tested the statistical significance of the interactions using the Wald test.

Of the participants, 60% had complete data on lifetime PA activity (age 30 and 50 years, and current age), 20% had one missing value (one of three time periods not reported), 10% had two missing values and only 10% had all three missing values. The proportion of incomplete data was 21% for waist–hip ratio and less than 5% for the remaining covariate data. A complete-subjects approach reduced the analytic cohort to 28 515 men, in which there were 1709 incidences of prostate cancer and 100 fatal prostate cancer cases. To evaluate a potential effect of missing values on the observed results, we used multivariate imputation by chained equations to obtain five imputed data sets of the analytic cohort including 45 887 men (van Buuren *et al*, 1999; Royston, 2004). The rate ratios estimated on imputed data sets were pooled together using Rubin's rule to obtain valid statistical inferences (Rubin and Schenker, 1986). We compared the rate ratios based on the two approaches (complete-subjects and multiple imputation) by calculating the relative difference defined as (RR complete case−RR multiple imputation)/RR multiple imputation.

All reported *P*-values are two-sided. All statistical analyses were performed with Stata, version 10 (StataCorp, College Station, TX, USA).

## Results

Baseline characteristics of the study population by quartiles of lifetime total PA are shown in Table 1. Compared with men in the bottom quartile of total activity, those in the higher quartiles were more likely to avoid sitting most of the time during their main work or occupation, more likely to walk or bike more than 60 min per day and less likely to have post-secondary education. Prostate cancer cases had an average age of 66 years at baseline and 72 years at diagnosis. The majority of cases (80%) were diagnosed because of clinical symptoms followed by health checkups (20%). Age and multivariate-adjusted rate ratios for prostate cancer incidence (total, localised and advanced) and mortality according to quartiles of lifetime average total PA are shown in Table 2.

**Figure 1:**
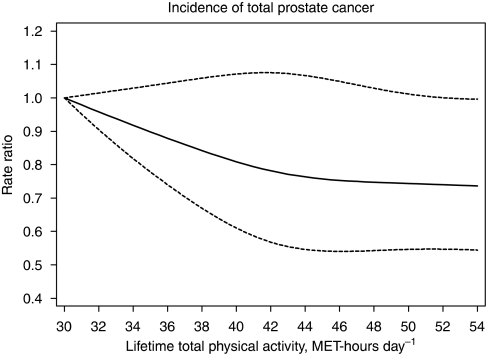
Multivariate rate ratio for lifetime average total physical activity (average of age 30 and 50 years, and baseline age) as predictor of total incidence of prostate cancer rates. Data were fitted using a Cox regression model with restricted cubic splines. Data were adjusted for baseline age, waist–hip ratio, height, diabetes, alcohol consumption, smoking status, years of education, total energy intake, consumption of dairy product and red meat and parental history with respect to prostate cancer. Dotted lines represent 95% confidence limits.

Lifetime total PA was significantly inversely associated with rates of total prostate cancer incidence. The age-adjusted rate ratio for the top quartile of lifetime total PA was associated with 17% lower risk of total prostate cancer compared with the bottom quartile. Further adjustment for waist–hip ratio, height, history of diabetes, alcohol consumption, smoking status, educational level, total energy intake, consumption of dairy product and meat and parental history of prostate cancer did not substantially change the estimate; incidence in the top quartile of lifetime total PA decreased by 16% (95% CI=2–27%) compared with the bottom. The inverse relationship between lifetime total PA modelled as a continuous variable and total prostate cancer risk is presented graphically in Figure 1. Excluding the first 4 years of follow-up did not change this association with PA; the multivariate-adjusted rate ratio in the top quartile of total PA significantly decreased by 17% (95% CI=1–30%) compared with the bottom.

The magnitude and direction of the estimates based on complete subjects and multiple imputation were overall similar. The multivariate-adjusted rate ratio based on five imputed data sets in the top quartile of total PA significantly decreased by 14% (95% CI=2–25%) compared with the bottom. The averages of the relative differences were 0.7% for the incidence of total prostate cancer, 0.2% for localised, 1.1% for advanced and 13.5% for fatal. In addition, we examined whether the influence of lifetime total PA on incidence differed according to the interval between study entry and diagnosis; no significant effect modification was observed (P_*interaction*_=0.30). Furthermore, there was no evidence of a significant interaction between lifetime total PA and age (*P*_*interaction*_=0.61), educational level (*P*_*interaction*_=0.28) or waist–hip ratio (*P*_*interaction*_=0.80).

For subtypes (Table 2), the multivariate-adjusted rate ratio in the top quartile of lifetime total PA was 18% lower (95% CI=0.66–1.03) for localised and 25% lower (95% CI=0.58–0.98) for advanced prostate cancer compared with the bottom quartile.

We then investigated the mutually adjusted effect of lifetime work or occupational activity and leisure-time walking or bicycling – the main determinants of the total PA score – on prostate cancer risk. Compared with men who mostly sit during their main work or occupation and controlling for walking or bicycling levels, men who sit half of the time experienced a 20% lower risk (95% CI=7–31%) of prostate cancer (Table 3). Heavy manual occupations were associated with a significantly lower risk of 28% (95% CI=10–43%) compared with sedentary work or occupation. The multivariate-adjusted (including work or occupation activity) incidence for those men walking or bicycling a lifetime average duration of over 60 min per day was 14% (95% CI=2–24%) lower than in those who walked or biked 20–40 min per day (Table 4). Advanced prostate cancer incidence decreased by 26% (95% CI=8–41%) for men walking or bicycling more than 60 min per day compared with those who walked or biked 20–40 min per day.

**Figure 2:**
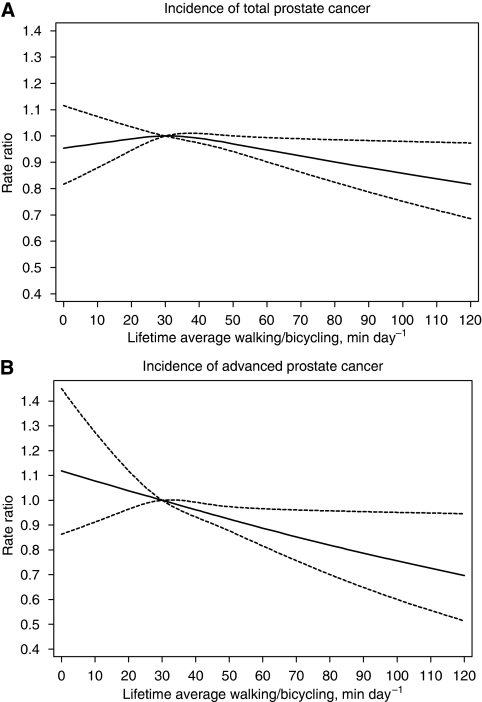
Multivariate rate ratio for lifetime average walking or bicycling duration (average of age 30 and 50 years, and baseline age) as predictor of total (**A**) and advanced (**B**) prostate cancer rates. Data were fitted using a Cox regression model with restricted cubic splines (reference value at 30 min per day). Data were adjusted for baseline age, lifetime work or occupational activity, waist–hip ratio, height, diabetes, alcohol consumption, smoking status, years of education, total energy intake, consumption of dairy product and red meat and parental history with respect to prostate cancer. Dotted lines represent 95% confidence limits.

Examining the associations with lifetime average walking or bicycling duration as a continuous variable, and using a reference value of 30 min per day, the adjusted rate ratio for total prostate cancer decreased linearly by 7% (95% CI=1–12%) for every 30 min per day increment in the range of 30–120 min per day. No significant trend was observed in the incidence of total prostate cancer below a lifetime average walking or bicycling of 30 min per day (Figure 2A).

**Figure 3:**
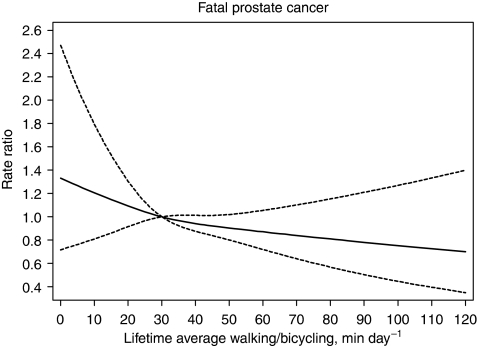
Multivariate rate ratios for lifetime average walking or bicycling duration (average of age 30 and 50 years, and baseline age) as predictor of fatal prostate cancer. Data were fitted using a Cox regression model with restricted cubic splines (reference value at 30 min per day). Data were adjusted for baseline age, lifetime work or occupational activity, waist–hip ratio, height, diabetes, alcohol consumption, smoking status, years of education, total energy intake, consumption of dairy product and red meat and parental history with respect to prostate cancer. Dotted lines represent 95% confidence limits.

Compared with men who walked or biked a lifetime average of 30 min per day, the adjusted rate ratio for localised prostate cancer linearly decreased by a marginally significant 8% (95% CI=0–16%) for every 30 min per day increment of lifetime average walking or bicycling in the range of 30–120 min per day. The adjusted rate ratio for advanced disease linearly decreased by 12% (95% CI=2–20%) for every 30 min per day increment in the range of 30–120 min per day (Figure 2B). For fatal prostate cancer, apart from the greater uncertainty of estimates due to the smaller number of cases, the results were similar to those for advanced disease (Figure 3). The fatality rate among those men who hardly ever walked or biked increased by about two-fold (rate ratio was 1.85; 95% CI=0.89–3.86) compared with men in the highest average lifetime walking or bicycling of 120 min per day, although this increased rate was not significant.

## Discussion

In this large population-based prospective cohort study of middle-aged and elderly men, we observed a significant inverse dose–response association between adult lifetime total PA and occupational and leisure-time walking or bicycling with prostate cancer incidence. Compared with those who mostly sit during their main work or occupation, men who sit half of the time or even less experienced a 20% lower risk of prostate cancer. Compared with men who walked or biked an average of 30 min per day, every increment of 30 min per day was associated with an incidence reduction of 7% for total, 8% for localised and 12% for advanced disease. No significant changes in incidence were observed below the lifetime walking or bicycling average duration of 30 min per day. Fatal prostate cancer rate was about two-fold higher among men who hardly ever walked or biked compared with those men who maintained the highest lifetime average of 120 min per day, although this increase was not statistically significant.

Our finding that the highest level of lifetime walking or bicycling, averaged approximately more than 40 years before diagnosis, was associated with 16% reduced risk is consistent with a previous Canadian case–control study that reported a non-significant 20% reduced risk of all prostate cancer for the top lifetime recreational level (Friedenreich *et al*, 2004). Our finding of an inverse association between lifetime walking or bicycling for 1 h per day or more and prostate cancer incidence supports the PA recommendation of the World Cancer Research Fund/American Institute for Cancer Research, which calls for a moderate activity of longer duration, namely, 60 min per day or more (WCRF/AICR, 2007). Furthermore, our finding of a strong inverse association (26% risk reduction) between lifetime walking or bicycling for an average of more than 1 h per day and advanced prostate cancer supports the findings of a previous large prospective cohort study of American men (Patel *et al*, 2005). In the American Cancer Society Cancer Prevention Study II Nutrition Cohort, baseline recreational PA (recent-past only) corresponding to 35 MET-hours per week or more (roughly corresponding to 1 h of walking or bicycling per day or more) was associated with a significant 31% risk reduction for aggressive prostate cancer (Patel *et al*, 2005).

In the Health Professional follow-up study, a strong inverse association (around 70% risk reduction) between recent-past vigorous PA and metastatic prostate cancer was observed only in men aged 65 years or older, with an evidence of effect modification by age (Giovannucci *et al*, 2005). In our analysis, we found no evidence of heterogeneity in the relationship across subgroups defined by age, educational level and waist–hip ratio.

The biological mechanisms by which PA may decrease prostate cancer risk are unknown, but PA may affect certain hormones hypothesised to be associated with prostate carcinogenesis, including insulin resistance (Goodyear and Kahn, 1998), adiponectin levels (Kelesidis *et al*, 2006; Bluher *et al*, 2007), insulin-like growth factors (Chan *et al*, 1998) and testosterone (Eaton *et al*, 1999).

Major strengths of our study include the large size of the cohort, its population-based and prospective design, the relatively large number of incident prostate cancers and the completeness of case ascertainment through the Regional and National Cancer Register. These features of the study substantially reduce the potential risk of recall and selection biases, and, importantly, increase the generalisability of the study findings. According to the National Prostate Cancer Register data, the most common cause of diagnosis in Sweden was clinical symptom (about 80% in our data), followed by health checkup (Adolfsson *et al*, 2007). Prostate-specific antigen testing may be considered to introduce bias, as this screening technique may only detect certain types of tumour. There is no official recommendation in Sweden on PSA testing as part of health checkups or for screening purposes in men without lower urinary tract symptoms (Adolfsson *et al*, 2007), hence any bias that may be introduced by PSA is of limited relevance in our data.

A potential limitation of this study is that PA was assessed through a self-administered questionnaire, which could lead to classification errors. Although our earlier validation study had indicated an overall good validity and reproducibility, there were some differences in measurement error that were dependent on individual characteristics such as body mass index (Norman *et al*, 2001). We found that the reliability of the historical PA questionnaire was relatively high (Spearman–Brown reliability were 0.7 at both age 50 and 30 years) (Orsini *et al*, 2007). Validity measures of recalled PA in the distant past were not available. However, a study on the quality of recall of PA in the distant past (32–35 years) found that about 70% of the participants (mean age 58 *vs* 60 years in our study) were good recallers, and quality was not significantly associated with age or body weight (Falkner *et al*, 2001).

As information on exposures was collected prospectively, any non-differential misclassification would probably have attenuated rather than exaggerated any true relationships, it is unlikely to explain the significant associations observed. Our study was observational, hence we cannot entirely rule out the possibility of residual confounding. Nevertheless, age-adjusted and multivariate-adjusted analyses provided overall similar results, suggesting that this is unlikely to explain our findings.

The inverse relation with PA might be the result of reverse causation if low PA was a result of an undiagnosed prostate cancer. If this were true, we might expect the effect of PA on prostate cancer risk to change after excluding the first 4 years of follow-up, but it did not.

Missing data related to prostate cancer or both PA and prostate cancer may lead to biased estimates (Demissie *et al*, 2003). However, we observed only relatively small differences when comparing complete-subject and multiple imputation approaches, suggesting that the subsample with complete data was a random subset of the entire sample.

Findings from this population-based prospective cohort study show that not sitting for most of the time during work or occupational activity and longer daily durations of the main component of active living (walking or bicycling) may be associated with reduced prostate cancer incidence. Our findings, which may have major public health implications in the prevention of prostate cancer, require confirmation by other well-designed studies.

## Conflict of interest

The authors declare no conflict of interest.

## Figures and Tables

**Table 1:** Age-standardized baseline characteristics by quartiles of lifetime (age 30 and 50 years, and current age) average total physical activity in the cohort of 45 887 Swedish men aged 45–79 years followed-up from 1998 to 2007

	**Quartiles of total physical activity, range (median), MET-h per day** a				
**Characteristics** b	**Q1, <39 (37)**	**Q2, 39–42.4 (41)**	**Q3, 42.5–46 (44)**	**Q4, >46 (48)**	**Missing**
No. of individuals	9143	9143	9143	9143	9315
Not mostly sitting at work or occupation (%)	48	97	99	100	91
Walking or bicycling >60 min per day (%)	3	11	20	40	23
Age (mean, years)	57	60	60	61	64
Body mass index (kg m^−2^)	26	26	26	26	26
Height (cm)	178	178	177	177	176
Waist–hip ratio ⩾0.95 (%)	42	40	41	40	45
Prostate diagnosis by symptoms (%)	71	76	79	68	82
History of diabetes (%)	8	8	8	8	13
Family history of prostate cancer (%)	6	7	7	9	11
Alcohol consumption (never, %)	4	4	5	6	7
Smoking (never, %)	39	38	35	35	33
Education (>12 years, %)	32	21	11	5	11
Intake (mean)					
Calories per day	2609	2670	2746	2923	2611
Dairy product (times per day)^c^	5.3	5.4	5.7	6.2	5.4
Red meat (times per day)^d^	1.3	1.3	1.3	1.3	1.2

a All factors, except age, were directly standardized to the age distribution of the study participants.

b Lifetime total physical activity (home or household work, work or occupation, walking or bicycling, exercise, and watching TV or reading) is the average of daily activities at age 30 and 50 years, and current (baseline) age, and it is expressed in metabolic equivalents (METs).

c Dairy products indicates milk, cheese, yogurt, cream, and crème fraiche.

d Red meat indicates meatballs, pork, veal, sausage, and black pudding

**Table 2:** Age-adjusted and multivariate rate ratios for total, localised, advanced and fatal prostate cancer according to quartiles of lifetime (age 30 and 50 years, and current age) average total physical activity levels

	**Lifetime average total physical activity, MET-hours/day**				
	**Q1, <39 (37)**	**Q2, 39–42.4 (41)**	**Q3, 42.5–46 (44)**	**Q4, >46 (48)**	***P*-trend**
*Total-incidence prostate cancer*					
No. of cases	419	414	447	429	
Age-adjusted RR (95% CI)	1	0.86 (0.75–0.98)	0.91 (0.79–1.04)	0.83 (0.73–0.96)	0.030
Multivariate RR (95% CI)^a^	1	0.86 (0.75–0.99)	0.91 (0.79–1.05)	0.84 (0.73–0.98)	0.065
					
*Localised*					
No. of cases	188	162	156	182	
Age-adjusted RR (95% CI)	1	0.74 (0.60–0.91)	0.70 (0.56–0.86)	0.78 (0.64–0.96)	0.045
Multivariate RR (95% CI)^a^	1	0.75 (0.61–0.93)	0.73 (0.58–0.91)	0.82 (0.66–1.03)	0.184
					
*Advanced*					
No. of cases	133	155	172	126	
Age-adjusted RR (95% CI)	1	0.97 (0.77–1.23)	1.05 (0.84–1.32)	0.73 (0.57–0.94)	0.014
Multivariate RR (95% CI)^a^	1	0.98 (0.78–1.25)	1.07 (0.84–1.36)	0.75 (0.58–0.98)	0.035
					
*Fatal prostate cancer*					
No. of cases	20	25	27	28	
Age-adjusted RR (95% CI)	1	0.97 (0.54–1.75)	1.02 (0.57–1.82)	0.99 (0.56–1.78)	0.989
Multivariate RR (95% CI)^a^	1	0.96 (0.53–1.75)	1.02 (0.55–1.87)	0.98 (0.53–1.83)	0.999

Abbreviations: CI, confidence interval; RR, rate ratio.

a Multivariate RRs were adjusted for age (years, continuous), waist–hip ratio (quartiles), height (continuous), diabetes (yes or no), alcohol consumption (current drinker, former drinker and never drinker), smoking status (current smoker, former smoker and never smoked), years of education (1–9 years, 9–12 years, more than 12 years), total energy intake (calories, continuous), consumption of dairy product (times per day, continuous) and red meat (times per day, continuous) and parental history with respect to prostate cancer (yes, no or not known). A complete-subjects analysis automatically discarded missing values on any covariate.

**Table 3:** Age-adjusted and multivariate rate ratios for total, localised, advanced and fatal prostate cancer according to lifetime (age 30 and 50 years and current age) work or occupational activity levels

	**Lifetime average work or occupational activity levels**				
	**Mostly sitting**	**Sitting half of the time**	**Mostly standing**	**Heavy manual labour**	***P*-trend**
*Total-incidence prostate cancer*					
No. of cases	291	405	1141	111	
Age-adjusted RR (95% CI)	1	0.81 (0.69–0.94)	0.80 (0.70–0.91)	0.72 (0.58–0.89)	0.003
Multivariate RR (95% CI)^a^	1	0.80 (0.69–0.93)	0.79 (0.69–0.91)	0.72 (0.57–0.90)	0.007
					
*Localised*					
No. of cases	123	184	439	36	
Age-adjusted RR (95% CI)	1	0.86 (0.68–1.08)	0.71 (0.58–0.87)	0.55 (0.38–0.79)	<0.001
Multivariate RR (95% CI)^a^	1	0.87 (0.69–1.09)	0.72 (0.58–0.90)	0.55 (0.38–0.82)	<0.001
					
*Advanced*					
No. of cases	96	129	422	39	
Age-adjusted RR (95% CI)	1	0.75 (0.57–0.97)	0.85 (0.68–1.07)	0.75 (0.52–1.10)	0.546
Multivariate RR (95% CI)^a^	1	0.74 (0.56–0.97)	0.88 (0.69–1.12)	0.79 (0.54–1.18)	0.853
					
*Fatal prostate cancer*					
No. of cases	16	21	82	8	
Age-adjusted RR (95% CI)	1	0.65 (0.34–1.25)	0.86 (0.50–1.48)	0.84 (0.36–1.97)	0.814
Multivariate RR (95% CI)^a^	1	0.64 (0.33–1.23)	0.88 (0.49–1.58)	0.89 (0.36–2.17)	0.679

Abbreviations: CI, confidence interval; RR, rate ratio.

a Multivariate RRs were adjusted for lifetime average walking or bicycling levels (hardly ever, <20, 20–40, 41–60 and >60 min per day), age (years, continuous), waist–hip ratio (quartiles), height (continuous), diabetes (yes or no), alcohol consumption (current drinker, former drinker and never drinker), smoking status (current smoker, former smoker and never smoked), years of education (1–9 years, 9–12 years, more than 12 years), total energy intake (calories, continuous), consumption of dairy product (times per day, continuous) and red meat (times per day, continuous) and parental history with respect to prostate cancer (yes, no or not known). A complete-subjects analysis automatically discarded missing values on any covariate.

**Table 4:** Age-adjusted and multivariate rate ratios for total, localised, advanced and fatal prostate cancer according to lifetime (age 30 and 50 years, and current age) walking or bicycling levels

	**Lifetime average walking or bicycling, min per day**					
	**Hardly ever**	**<20**	**20–40**	**41–60**	**>60**	***P*-trend**
*Total-incidence prostate cancer*						
No. of cases	55	391	706	411	403	
Age-adjusted RR (95% CI)	0.99 (0.75–1.30)	0.97 (0.86–1.10)	1	0.96 (0.85–1.09)	0.87 (0.77–0.98)	0.049
Multivariate RR (95% CI)^a^	1.03 (0.78–1.36)	0.97 (0.86–1.10)	1	0.96 (0.85–1.08)	0.86 (0.76–0.98)	0.028
						
*Localised*						
No. of cases	21	142	293	179	161	
Age-adjusted RR (95% CI)	0.92 (0.59–1.43)	0.86 (0.70–1.05)	1	1.01 (0.84–1.22)	0.84 (0.69–1.02)	0.447
Multivariate RR (95% CI)^a^	0.98 (0.63–1.53)	0.86 (0.70–1.05)	1	1.00 (0.83–1.21)	0.84 (0.69–1.02)	0.393
						
*Advanced*						
No. of cases	15	149	248	148	128	
Age-adjusted RR (95% CI)	0.85 (0.50–1.44)	1.09 (0.89–1.34)	1	0.94 (0.77–1.16)	0.74 (0.60–0.92)	0.002
Multivariate RR (95% CI)^a^	0.88 (0.52–1.49)	1.10 (0.89–1.35)	1	0.94 (0.76–1.15)	0.74 (0.59–0.92)	0.001
						
*Fatal prostate cancer*						
No. of cases	4	26	44	28	25	
Age-adjusted RR (95% CI)	1.71 (0.61–4.81)	1.19 (0.73–1.93)	1	0.93 (0.58–1.49)	0.73 (0.45–1.20)	0.062
Multivariate RR (95% CI)^a^	1.81 (0.64–5.12)	1.21 (0.74–1.97)	1	0.90 (0.56–1.46)	0.72 (0.44–1.18)	0.044

Abbreviations: CI, confidence interval; RR, rate ratio.

a Multivariate RRs were adjusted for lifetime work or occupational activity levels (mostly sitting, sitting half of the time, mostly standing and heavy manual labour), age (years, continuous), waist–hip ratio (quartiles), height (continuous), diabetes (yes or no), alcohol consumption (current drinker, former drinker and never drinker), smoking status (current smoker, former smoker and never smoked), years of education (1–9 years, 9–12 years, more than 12 years), total energy intake (calories, continuous), consumption of dairy product (times per day, continuous) and red meat (times per day, continuous) and parental history with respect to prostate cancer (yes, no or not known). A complete-subjects analysis automatically discarded missing values on any covariate.
